# Finnish physicians’ stress related to information systems keeps increasing: a longitudinal three-wave survey study

**DOI:** 10.1186/s12911-017-0545-y

**Published:** 2017-10-17

**Authors:** Tarja Heponiemi, Hannele Hyppönen, Tuulikki Vehko, Sari Kujala, Anna-Mari Aalto, Jukka Vänskä, Marko Elovainio

**Affiliations:** 1 National Institute for Health and Welfare, P.O. Box 30, 00271 Helsinki, Finland; 2 Aalto university, Espoo, Finland; 3 Finnish Medical Association, Helsinki, Finland; 4 University of Helsinki, Helsinki, Finland

**Keywords:** Information systems, Physicians, Stress, Electronic health records, Longitudinal research

## Abstract

**Background:**

Poorly functioning, time-consuming, and inadequate information systems are among the most important work-related psychosocial factors causing stress in physicians. The present study examined the trend in the perceived stress that was related to information systems (SRIS) among Finnish physicians during a nine-year follow-up. In addition, we examined the associations of gender, age, employment sector, specialization status, leadership position, on-call burden, and time pressure with SRIS change and levels.

**Methods:**

A longitudinal design with three survey data collection waves (2006, 2010 and 2015) based on a random sample of Finnish physicians in 2006 was used. The study sample included 1095 physicians (62.3% women, mean age 54.4 years) who provided data on SRIS in every wave. GLM repeated measures analyses were used to examine the associations between independent variables and the SRIS trend during the years 2006, 2010, and 2015.

**Results:**

SRIS increased during the study period. The estimated marginal mean of SRIS in 2006 was 2.80 (95% CI = 2.68–2.92) and the mean increase was 0.46 (95% CI = 0.30–0.61) points from 2006 to 2010 and 0.25 (95% CI = 0.11–0.39) points from 2010 to 2015. Moreover, our results show that the increase was most pronounced in primary care, whereas in hospitals SRIS did not increase between 2010 and 2015. SRIS increased more among those in a leadership position. On-call duties and high time-pressures were associated with higher SRIS levels during all waves.

**Conclusions:**

Changing, difficult, and poorly functioning information systems (IS) are a prominent source of stress among Finnish physicians and this perceived stress continues to increase. Organizations should implement arrangements to ease stress stemming from IS especially for those with a high workload and on-call or leadership duties. To decrease IS-related stress, it would be important to study in more detail the main IS factors that contribute to SRIS. Earlier studies indicate that the usability and stability of information systems as well as end-user involvement in system development and work-procedure planning may be significant factors.

## Background

The most stressful work-related factors among physicians have traditionally been time pressure, work load, difficult patients, and problems in team work [1–3]. Recently, however, poorly functioning, time-consuming, and inadequate information systems (IS) have emerged as one of the most stressing factors in physicians’ work [4, 5]. Moreover, it has been shown that stress that is related to information systems (SRIS) has increased in the period 2006 to 2010 [6]. The use of IS has been found to increase physicians’ workload [7] and cognitive demands [8]. The resulting information chaos may have ramifications, for example, for physician performance and patient safety [9].

The increased number of functions in electronic health records (EHRs) has been associated with more stress and less job satisfaction [10]. In addition, time pressure was more strongly related to negative outcomes such as burnout, dissatisfaction, and intent to leave among those physicians who had to manage a high number of EHR functions compared to those managing a low number of functions [10]. Poor EHR usability, time-consuming data entry, interference with face-to-face patient care, inability to exchange health information between health information systems (HIS), and impairments in clinical documentation have been found to be prominent sources of physicians’ professional dissatisfaction [11].

The traditional doctor–patient relationship has been impacted by the use of HIS. Physicians have to turn to the computer to complete electronic forms during the encounter, and this can be time consuming, especially if the physician suffers from limited computer skills. For some physicians, aspects of EHRs represent a distraction during visits [12]. In a US study, the average screen gaze time of physicians ranged from 25% to 55% of the consultancy session, inevitably meaning less eye-contact and less conversation with the patient [13]. In the same study, 92% of physicians felt that engagement with electronic medical records (EMR) disturbed communication with their patients. Screen gaze has been found to be particularly disruptive to psychosocial inquiry and emotional responsiveness, indicating that visual attentiveness to the monitor rather than eye contact with the patient may inhibit sensitive or full patient disclosure [14]. It has been found that after implementation of an EHR, the physician’s time in the clinical setting has transferred from directly caring for patients to documenting in the EHR [15]. Physicians’ have been rated as having less effective communication when they spent more time looking at the computer and when there were more periods of silence in the consultation [16].

EHRs may be challenging to use because of the multiplicity of screens, options, and navigational aids [17]. However, the demands and pressures of care may not allow physicians time to master all the complex system functions [18]. Physicians may also see it as a burden if forced to learn how to use the EHR system effectively and efficiently. It is also possible that a lack of appropriate skills and time to learn them lead physicians to regard the EHR system as extremely complicated.

In addition, the ever-changing new functionalities and systems require constant development of physicians’ skills. In Finland in 2014, only 24% of physicians in health centres and 37% in hospitals thought that HIS did not require long orientation and only half of the physicians knew where to give feedback about HIS problems [19]. These ratings worsened after the year 2010. Many physicians complain about poor service from the information system vendor, including a lack of training and support for problems [20]. However, IS changes may also be a positive improvement, which might help to decrease stress levels related to IS.

### The Finnish context

Finnish public health care is mainly financed through taxation. All residents in Finland have a right to use public health care services including primary health care and specialized health care. Provision of health care services is mainly in responsibility of municipalities. All Finnish residents have a National Health Insurance coverage partly reimbursing also the costs coming from the use of private health services. The private sector consists mainly on a) customers themselves paying and purchasing their care or by using health insurances, and b) occupational health services where employers pay services for their employees. Private health care sector use has increased from 2000 to 2009, though in the last few years, the trend has been declining; in 2013, the private sector constituted 5.9% of total health expenditure [21]. Some municipalities have outsourced parts or all of their health centres through open tendering. However, most of services are still provided by municipalities.

HIS have undergone notable recent reforms in Finland, adding to the burden of dealing with novel functionalities and systems. The public sector EHR coverage in Finland reached 100% in 2010, while almost every private sector provider also uses an EHR system [21]. The EHR infrastructure is not uniform, however, though the number of trade names has decreased and since 2014, there have been five different trade names operating in public secondary care and six in public primary care [21]. In a move towards integrated patient data services, Finland has launched the national digital repository for electronic patient data, Kanta, targeted to health care service providers, pharmacies, and citizens, which has been deployed in phases throughout Finland during the period 2012–2017. Kanta services include electronic prescription, My Kanta pages for citizens, a patient data repository, and a pharmaceutical database. Joining the Kanta services is mandatory for all public health care providers, while private service providers that use electronic documentation also have to join the Kanta services. By the end of 2014, all pharmacies and public service providers with the exception of one had joined the national ePrescription service [21]. At that time, a large proportion of private sector providers had also joined, and the national ePrescription system was almost fully implemented. From the beginning of 2017, ePrescribing was the only and obligatory means for prescribing and dispensing medications.

### Aims of the study

The present study aimed to examine the 9-year longitudinal development of SRIS levels among Finnish physicians. SRIS levels were examined in three waves in the years 2006, 2010 and 2015. Thus, the present study adds to the previous literature by giving valuable longitudinal information on how physicians experience IS and how stressful experiences have developed both recently and over the 9-year period.

Given that previous research has shown that age, gender, employment sector, and specialty may have an effect on EHR adoption and attitudes towards EHRs [22–25], we also examined the effects of these factors on the levels and development of SRIS over the study period. Moreover, the use of IS may lead to information chaos, which is known to be influenced by mental workload and time available to cope with this information chaos [9]. Therefore, we also examined the effects of challenges at work, such as time pressures, on-call burden, and leadership position, on levels and development of SRIS over time. Thus, the present study also adds to previous research by examining possible work-related correlates of SRIS.

## Methods

### Study sample

The present study is a part of the Finnish Health Care Professionals Study that started in 2006. The data were gathered from a random sample of 5000 physicians in Finland (30% of the physician population) based on the database of physicians maintained by the Finnish Medical Association. The register covered all licensed physicians in Finland. In wave 1 (2006), data were gathered via postal questionnaires. Non-respondents were sent a reminder and a copy of the questionnaire up to two times. Responses were received from 2841 physicians (response rate 57%). The sample was representative of the eligible population in terms of age, gender, and employment sector [26]. Ethical approval for the study was obtained from the Ethical Review Board of the National Institute for Health and Welfare.

Four years later, in wave 2 (2010), data were gathered via either a web-based or traditional postal survey. In wave 1, respondents were asked for their consent to participate in follow-up surveys, with 2206 agreeing to participate in future surveys. Those who had died or had incorrect address information were excluded (*n* = 37). Thus, in wave 2, the follow-up survey was sent to 2169 physicians. First, an email invitation to participate in the web-based survey was sent, which was followed by two email reminders. For those who did not respond to these, a postal questionnaire was sent once. Email and postal addresses were obtained from the Finnish Medical Association. The total number of respondents was 1705 (response rate 79%; 60% women).

In wave 3 (2015), data were gathered either via a web-based or traditional postal survey. Questionnaires were sent to those that gave consent for follow-up in the 2006 survey. Those who had died during the follow-up or who had an unknown address in 2015 (*n* = 47) were excluded, leaving 2159 physicians. Of these 1462 physicians responded (response rate 68.3%). The present study uses a subsample that includes 1095 physicians (62.3% women, mean age 54.4, SD = 9.0, age range 34–72) who had answered the SRIS survey items in every wave. The present sample included more women (57.4% in eligible population), slightly older respondents (mean age 47.3 in eligible population), and more specialists (66.8% in our sample vs. 61.6 in eligible population) compared to the eligible population.

### Measurements

*Stress related to information systems (SRIS)* was measured with two items asking “How often have you been distracted, worried, or stressed about (during the past half-year period) a) constantly changing information systems and b) difficult, poorly performing IT equipment / software.” The items were rated on a 5-point Likert-scale ranging from 1 (*never*) to 5 (*very often*) with higher scores indicating higher SRIS. A mean value for the two items was calculated, with the reliability (Cronbach’s alpha) of this composite scale in the present sample being 0.84 in 2006, 0.84 in 2010, and 0.85 in 2015.

*Employment sector* was categorized into four groups in the analyses: a) those who worked in primary care in every wave (*n* = 162), b) those who worked in hospitals in every wave (*n* = 343), c) those who worked in the private sector in every wave (*n* = 102), and d) others (*n* = 466). *Specialization status* was used from the first wave in 2006 and it was categorized as a) not specialized, b) specialization ongoing, and c) specialists.

*Leadership position* was categorized into three groups: a) those who had a leadership position in every wave (*n* = 166), b) those who were not in a leadership position in any wave (*n* = 559), and c) others (*n* = 323).

*On-call burden* was categorized into three groups: a) those who had on-call duties in every wave (*n* = 318), b) those who were not on-call in any wave (*n* = 431), and c) others (*n* = 334).

*Time pressure* was measured with three items that were developed based on previous research among nurses and health care staff and which have shown adequate psychometric properties [27]. The time-pressure scale measures stress due to time shortages at work and scheduling problems. An example item: “How often have you been distracted from, worried about, or stressed about (during the past half-year period) not being able to do your work properly.” The items were rated on a 5-point Likert-scale ranging from 1 (*never*) to 5 (*very often*), with higher scores indicating higher time pressure. A mean value of the three items was calculated and the reliability of the composite scale in the present sample was 0.84 in 2006, 0.87 in 2010, and 0.87 in 2015. For the purpose of analyses, time pressure scores were categorized into three groups: a) those who had high levels of time pressure in every wave (above the median every time; *n* = 262), b) those who had low levels of time pressure in every wave (below the median every time; *n* = 296), and c) others (*n* = 534).

These above mentioned aggregated groups regarding sector, leadership position, on-call duties and time pressure were used for statistical analyses to get a measure of cumulated exposure over time.

### Statistical analysis

GLM repeated measures analysis was performed to examine the effects of independent variables (gender, age, specialization status, employment sector, leadership position, on-call burden, and time pressure) on the development of SRIS over the study period. The associations of Mauchly’s Test of Sphericity indicated that the assumption of sphericity had been violated, (*p* < .001), and therefore, a Greenhouse-Geisser correction was used. All analyses were conducted using the SPSS statistical package 23.0.

## Results

The characteristics of the study sample are reported in Table 1. The majority of respondents were specialized already in 2006 and in 2015 the number of specialists had further increased. The number of private physicians had increased from 12% in 2006 to 22% in 2015, whereas the numbers of primary care physicians and hospital physicians had slightly decreased. The proportion of those who had a leadership position had slightly increased from 2006 to 2015. In contrast, the proportion of those who had on-call duties had decreased from 2006 to 2015. Time pressure had decreased during the study period (F = 54.1, *p* < 0.001).

**Table 1:** Characteristics of the study sample

	2006		2010		2015		Whole period^a^	
	n	%	n	%	n	%	n	%
Specialization status								
Not specialized	149	13.8	114	10.4	108	9.9		
Specialization on-going	209	19.4	117	10.7	43	3.9		
Specialists	721	66.8	862	78.9	934	85.6		
Sector								
Primary care	240	22.1	244	22.5	217	19.8	162	15.1
Hospital	482	44.5	479	44.2	443	40.5	343	32.0
Private	134	12.4	188	17.3	238	21.8	102	9.5
Other	228	21.0	173	16.0	196	17.9		
Leadership position								
Yes	305	28.5	345	31.6	343	32.0	166	15.2
No	767	71.5	746	68.4	728	68.0	559	51.1
On-call duties								
Yes	586	53.7	471	43.1	389	35.7	318	29.0
No	505	46.3	622	56.9	700	64.3	431	39.4
	Mean	SD	Mean	SD	Mean	SD		
SRIS	2.93	1.2	3.31	1.1	3.48	1.1		
Time pressure	3.36	1.0	3.18	1.0	3.06	1.1		

*SRIS* stress related to information systems

^a^Aggregated frequencies showing those who were in the category in every measurement phase

The results of the GLM repeated measures analysis showed that there was a significant effect of time on SRIS (F *=* 7.15*, p* = .001), indicating that SRIS had increased during the study period. Post hoc tests using the Bonferroni correction revealed that estimated marginal means of SRIS starting from 2.80 (95% CI = 2.68–2.92) in 2006 increased by an average of 0.46 (95% CI = 0.30–0.61) points from year 2006 to 2010 (*p* < 0.001) and then increased by an additional 0.25 (95% CI = 0.11–0.39) points between years 2010 and 2015 (p < 0.001).

**Fig. 1:**
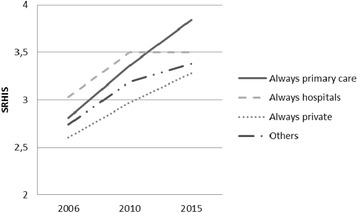
The levels of stress related to information systems (SRIS) according to employment sector

Working/health-care sector had a significant interaction with time in relation to SRIS (F = 3.74, *p* = 0.001). Those who had worked in primary care at all time points had the highest increase in SRIS from 2006 to 2015 (Fig. 1). Those who had worked in hospitals had the highest levels of SRIS in the years 2006 and 2010, but in 2015 the SRIS levels had not increased further. Among private sector physicians, the SRIS levels had increased over the waves, but were less pronounced than in other sectors.

**Fig. 2:**
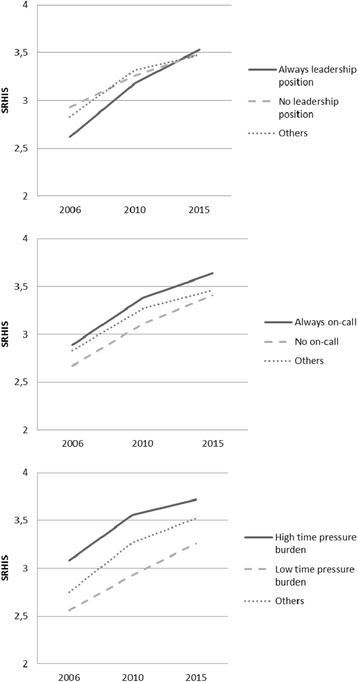
The levels of stress related to information systems (SRIS) according to leadership position, on-call burden and time pressure burden

Leadership position had a significant interaction with time in relation to SRIS (F = 2.80, *p* = 0.024). The highest increase of SRIS was among those who were in a leadership position in every wave; in 2006 they had the lowest levels of SRIS, but in 2015 the highest (Fig. 2). Those who did not have a leadership position at all had the highest levels in 2006, but their increase in SRIS was not so pronounced as for others. The effect of on-call burden did not vary across the different waves, but it had a significant between-subjects effect (F = 4.86, *p* = 0.008), indicating that those who had an on-call burden in every wave had higher levels of SRIS in every wave as well (Fig. 2). Similarly, the effect of time pressure did not vary across the years, but it had a significant between-subjects effect (F = 23.75, *p* < 0.001). Those who had high levels of time pressure in every wave also had high levels of SRIS in every wave (Fig. 2). Age was not related to SRIS.

## Discussion

The present 9-year longitudinal study with three waves shows that stress that was related to ever-changing, difficult, and poorly functioning information systems has increased among Finnish physicians between 2006 and 2015. Moreover, our results show that this increase was most pronounced in primary care, whereas in hospitals this increase had stopped between 2010 and 2015. Those who had a leadership position in every wave had a higher increase of SRIS than those who did not have a leadership position at all. The effects of burden coming from on-call duties and high time pressures did not vary across time: Those who had on-call duties or high time pressures in every wave also had higher levels of SRIS than their counterparts in every wave.

Our findings are in line with previous findings related to HIS showing that physicians suffer from strain and stress from poorly functioning and inadequate HIS [4–6, 19, 28]. Our results also show that IS-related stress keeps increasing. According to other studies, physicians complain about HIS that work too slowly and unreliably and that poorly support physicians’ daily work and multiprofessional co-operation [4, 28]. They also rate their EHR systems very critically, reporting several usability problems, system failures, and deficiencies as well as poor support for the documentation and retrieval of patient data [25, 29]. Poor EHR usability, time-consuming data entry, interference with face-to-face patient care, and inability to exchange health information have been associated with physicians’ professional dissatisfaction [11], while a higher number of EHR functions has been associated with stress and job dissatisfaction [10, 11].

However, even though physicians experience stress from problems associated with IS, previous studies have shown that they also acknowledge their value. For example, primary care physicians in Scotland considered that EHRs are an essential part of their work during a consultation and facilitate patient care and make information more accessible [30]. However, they pointed out issues that needed improving, such as system failures, information overload, difficulties in adjusting to new systems, interoperability problems, and poor usability. Swedish physicians regarded their EHR system as easy to use in general and for prescribing drugs, while believed ePrescriptions to be time saving and safer than handwritten prescriptions [31].

We showed that primary care physicians had the highest increase of SRIS from 2006 to 2015 and their levels of SRIS were highest in 2015. In contrast, hospital physicians had the highest levels in 2006 and 2010, but in 2015 their levels had not increased further from 2010 levels. Thus, our results suggest that in hospitals, the negative trend related to IS has levelled out. Previous studies with another sample have shown that in 2010 and in 2014, hospital physicians were most critical of HIS in Finland [4, 28]. Also results from the USA suggest that hospital physicians have worse attitudes about EMRs [32]. One reason for the levelling out of SRIS among Finnish hospital physicians might be that improvements in usability of the systems used in the hospitals may have been implemented. Moreover, the national information services platform (ePrescription and eArchive) have been implemented between 2010 and 2015, supporting medication management and summary views of patient data. It may also be that changes in the context of other than information technology (IT) have levelled out the impact of poor usability of IS in Finnish hospitals. The actual effect of IS on the levelling of SRIS in hospitals requires further examination and it would be important to obtain more information about which changes in IS are stressful and which are helpful. This seems to be a double-edged sword: On the one hand, changing systems are a source of stress, but on the other hand they may offer improvements and reduce strain.

Our finding that private physicians had the lowest levels of SRIS throughout the study period is in accordance with previous findings. A previous Finnish study found that private physicians are more satisfied with their electronic patient records (EPR) than public sector physicians [33]. Especially private sector physicians were more satisfied with the stability and speed of their EPRs, as well as experiencing less often endangering of patient safety related to EPRs. Compared to responses from the public sector (primary care and hospitals), Finnish physicians working in the private sector have been more satisfied with their EHR systems, specifically the user interface characteristics and support for routine tasks [25].

We found that constantly changing, poorly functioning, and difficult information systems are experienced as the most stressful when facing more other work-related challenges, such as high job-demands and a need to hurry. High time pressures and on-call burden were associated with high SRIS in all the study waves and leadership position in the last wave. Thus, the complexity, time-pressure, and distraction aspects of EHRs [12, 13, 15, 17] seem to be most strenuous when the work is already challenging and the physician has difficulties in coping with the work.

The present study was a 9-year longitudinal study with three measurement phases with an interval of 4–5 years. The doings and working places of the respondents between the study measurements is not possible to know. Thus, respondent may have held other positions between the measurements than during the measurement phases. Moreover, we used self-reported measures, and this may be associated with problems in inflation of the strengths of relationships and with common-method variance. Therefore, well-known validated measures showing good reliability were used. Our key limitation is that our main variable SRIS was a mean of only two items rather than on many elements. However, this variable showed good reliability (0.84–0.85) and has previously been widely used and associated, for example, with employees’ distress (General Health Questionnaire) and higher levels of on-call duties [34, 35].

Moreover, although we controlled for factors such as age, gender, and specialization, we cannot rule out the possibility of residual confounding. In addition, our sample is not completely representative of the present physician population in Finland. Our sample included more women, older physicians, and more specialists than the eligible population in 2015. Our findings should not be generalized to health care systems using different kinds of IT-systems or dissimilar styles of organizing health care.

## Conclusions

The present study found that poorly functioning IS are a prominent source of stress among Finnish physicians and this stress continues to increase. This is alarming particularly since SRIS has been associated with higher levels of distress, lower self-rated health, and lower work ability [35]. Thus, health organizations and software providers should take more seriously the problems with IS in health care.

It is alarming that stress levels due to IS continue to increase among physicians. IS have become a part of everyday life for physicians over a period of several years and previous studies suggest that with time and practice, the influence of poor usability will diminish [36]. In parallel to learning, one would assume that stress levels would also level out. The fact that stress levels still continue to rise implies that current information systems are too complicated, even after years of trying to learn, especially in the context of high time pressures. In Finland, several new systems have been adopted over the study period and stress cumulates when physicians have to get used to new systems before they have even become accustomed to previous systems.

We found support for the suggestion that high mental workload and lack of time to cope among physicians may have an effect on the ramifications of information chaos resulting from IS [9]. Thus, organizations should pay more attention to the overall strain that physicians experience. In addition, organizations should implement arrangements to ease the stress and extra duties coming from IS for those with high job strain, such as high workload and a lot of on-call or leadership duties.

However, the present study also found promising results, given that hospitals had been able to stem the increase in SRIS. Future studies should try to find IS and work-related factors that could help to ease the stress coming from poorly functioning IS in health care. Of course, it would be most important to improve the usability and stability of the systems, as well as to involve end-users in the development of HIS and in the planning of work procedures.

## Abbreviations

EHR: Electronic health record
EMR: Electronic medical record
EPR: Electronic patient record
HIS: Health information systems
IS: Information systems
IT: Information technology
SRIS: Stress related to information systems
